# Immature Platelet Fraction and Other Platelet Indices in Type 2 Diabetes Mellitus

**DOI:** 10.7759/cureus.79093

**Published:** 2025-02-16

**Authors:** Nauman A Siddiqui, Shiva Narang, Amitesh Aggarwal, Richa Gupta, Vrinda Goel

**Affiliations:** 1 Department of Medicine, University College of Medical Sciences, Guru Teg Bahadur Hospital, New Delhi, IND; 2 Department of Pathology, University College of Medical Sciences, Guru Teg Bahadur Hospital, New Delhi, IND; 3 Department of Cardiology, Govind Ballabh Pant Institute of Postgraduate Medical Education and Research, New Delhi, IND

**Keywords:** antiplatelet in diabetes, diabetes mellitus, immature platelet fraction, mean platelet volume, platelet and diabetes, plateletcrit, platelet distribution width, platelet indices

## Abstract

Background: Diabetes mellitus (DM) has become a significant global health concern, with its associated complications imposing a substantial burden on healthcare systems worldwide. While it is widely recognized as a complex metabolic disorder, the understanding of its pathophysiology continues to evolve, leading to the identification of various diabetes subtypes and their diverse clinical presentations. Despite extensive research, the impact of diabetes on platelet function remains a lesser explored domain.

Methods: We conducted a case-control study to investigate the variation of platelet indices in individuals with type 2 diabetes mellitus (T2DM) compared to healthy controls. Our study, conducted at a tertiary care facility in the national capital region, aimed to identify potential prognostic markers and explore the utility of platelet indices in predicting diabetic complications. A total of 230 participants, including 115 T2DM cases and 115 age and sex-matched healthy controls, were enrolled in the study. Baseline investigations, including hemogram and platelet indices, were performed and statistical analyses were conducted to compare the results between the two groups.

Results: Significant differences were found in platelet parameters between T2DM cases and controls, with elevated levels observed in individuals with T2DM. Immature platelet fraction (IPF), mean platelet volume, plateletcrit, platelet distribution width, platelet large cell count, and platelet large cell ratio were among the parameters that were significantly elevated in individuals with T2DM. Furthermore, IPF emerged as a promising biomarker, demonstrating excellent diagnostic performance with an area under the ROC curve of 0.941. A cutoff value of IPF (%) ≥9.6 showed high sensitivity (86%) and specificity (95%) in distinguishing T2DM cases from controls.

Conclusion: Our study provides novel insights into the relationship between platelet indices and T2DM, highlighting the potential utility of IPF as a prognostic marker in diabetic individuals. These findings warrant further investigation in larger cohorts and prospective studies to validate the proposed cutoff values and explore their clinical and therapeutic implications in the management of T2DM and delaying its complications.

## Introduction

Diabetes mellitus (DM) has emerged as a major health concern over the past few decades. Diabetes and its associated complications pose a huge burden on the healthcare system globally. It is a complex disease that is characterised by features of chronic hyperglycaemia, metabolic abnormalities and long-term macrovascular and microvascular complications which involve multiple organ systems including the blood vessels, eyes, kidneys and nerves to name a few. According to the most recent International Diabetes Federation (IDF) data, 537 million people, approximately 10% of all people aged 20-79 in the world, were living with diabetes as of 2021, which is expected to increase to 643 million by 2030 and to 783 million by 2045. Additionally, an estimated 240 million of those who have diabetes are predicted to be currently undiagnosed [1].

Decades of work on diabetes now classify it as a spectrum or a group of metabolic diseases marked by hyperglycaemia and its resulting complications among other things. This contrasts our conventional understanding of type 1 and type 2 diabetes mellitus. There are now various types of diabetes defined as per the recent guidelines. The presentation also varies from innocuous symptoms like frequent change of glasses to life-threatening complications like diabetic ketoacidosis. As per the American Diabetes Association (ADA), simple blood sugar levels are used for the diagnosis of diabetes [2]. Mass screening processes are included in various national guidelines to detect the asymptomatic population.

Diabetes is a heterogeneous complicated metabolic disorder that affects multiple organ systems in the body. Macro and microvascular complications lead to dysfunction in various systems, including the cardiovascular, nervous, renal, and haematological systems, all of which are affected to varying degrees in this disease process. Diabetes has a long natural history and presents at different ages in different patients. There is unpredictability of the development of complications as they are a function of the duration of disease and glycaemic control in the background of varied genetic makeup. The cornerstone to slowing this process is strict glucose control. Haematological parameters also do not remain untouched by persisting dysglycaemia, platelets being one of them.

The influences on haematological parameters are diverse including anaemia of chronic disease, anaemia due to therapy of diabetes, effect on immunity and so on. Hyperglycaemia affects the leucocytes due to various cytological processes making the person more susceptible to infections. However, little is known about the effects of diabetes on platelets. There is a large body of evidence that platelets are likely to contribute to inflammation in multiple diseases. They are also known to contribute towards accelerated atherosclerosis, especially in conditions like diabetes, hypertension, metabolic syndrome and other chronic inflammatory conditions. Platelet indices are a marker of platelet activation. They are related to platelet morphology and proliferation kinetics. They include Immature platelet fraction (IPF), mean platelet volume (MPV), platelet distribution width (PDW), platelet large cell count (PLCC), platelet large cell ratio (PLCR) and plateletcrit (PCT). Platelet activation plays a key role in atherothrombosis in type 2 diabetes mellitus (T2DM) and increased in vivo platelet activation with enhanced thromboxane biosynthesis has been reported in patients with impairment of glucose metabolism even in the early stages of the disease.

As the literature suggests the role of platelets in the development of diabetic complications, we planned to study the variation of platelet indices in type 2 diabetics and compare it with non-diabetics.

## Materials and methods

This case-control study was conducted in a tertiary care facility (Guru Teg Bahadur Hospital, University College of Medical Sciences, New Delhi) in the national capital region of India from September 2022 to February 2024. We aimed to compare platelet indices between diabetic and non-diabetic groups. We recruited known cases of T2DM between 30 and 59 years of age within three years of diagnosis, on oral hypoglycaemic agents (OHAs) or lifestyle modifications without complications, and age/sex-matched healthy controls. Exclusion criteria were T2DM patients on insulin therapy, those with known diabetic complications, and pregnant women. Baseline investigations included hemogram; liver function tests including total and direct bilirubin, serum glutamic-oxaloacetic transaminase, serum glutamic pyruvic transaminase and alkaline phosphatase; kidney function tests including blood urea and serum creatinine; urine routine examination including urine sugar, urine albumin, urine ketone and urine casts; serum lipids including total cholesterol, high-density lipid, very-low-density lipids and triglycerides; fasting blood sugar (FBG), postprandial blood sugar (PPBS), and HbA1c (glycated haemoglobin) for both groups, with additional fundus examination and ECG (electrocardiography) for cases. Blood samples were processed to estimate IPF and other platelet indices, with normal IPF reference ranges set at 1-7%. The estimated sample size was 115 subjects in each group. Data were analysed using IBM SPSS Statistics for Windows, Version 20 (Released 2011; IBM Corp., Armonk, New York, United States), with continuous data presented as mean (SD) or median (inter-quartile range) and categorical data as numbers and percentages. Normality was tested using the Kolmogorov-Smirnov test and Box-Whisker plots. Comparisons were made using unpaired t-tests for normally distributed biomarkers and Mann-Whitney U tests for non-normally distributed biomarkers. Pearson or Spearman correlation was used to assess association strength, while receiver operating characteristic (ROC) curves evaluated the classification strength of biomarkers and identified optimal cut-off points using the Youden index. Despite age and sex matching, independent parametric and non-parametric tests were applied to account for confounders, with baseline characteristics compared using Student’s t-test and the chi-square test. Ethical clearance was taken from the Institutional Ethics Committee for Human Research (IEC-HR), University College of Medical Sciences, University of Delhi (IECHR-2022-55-10).

## Results

A total of two hundred thirty participants (115 cases and 115 controls) were included. Participants were diagnosed with diabetes within the last three years and were either on lifestyle modifications or OHAs with no micro- or macrovascular complications. Among the demographic parameters and comorbidities, the diabetic group had a significantly higher number of participants with hypertension and hypothyroidism (Table 1).

**Table 1:** Demographic characteristics and comorbidities among cases and controls Categorical variables are expressed as counts (percentages) and p-values. Continuous variables are expressed as mean (± standard deviation) and p-values based on the t-test. Age is represented in years; * marked values have p-values <0.05, which is a statistically significant value. Values for age and BMI were calculated using the Wilcoxon-Mann-Whitney U test, whereas gender, thyroid status, and smoking were calculated using the chi-square test. BMI: body mass index

Characteristic	Cases (n = 115)	Matched Controls (n = 115)	Total (N=230)	Significance (p-value)	Test Value
Demographic characteristics					
Age (years)	48.78 (±6.91)	48.78 (±6.91)		1.0	6612.500
BMI (kg/m^2^)	24.46 (±2.42)	24.90 (±1.7)		0.113	5287.000
Sex				1.0	
Male	56 (48.7%)	56 (48.7%)	112 (48.7%)		0.000
Female	59 (51.3%)	59 (51.3%)	118 (51.3%)		
Smoking				0.78	
No	75 (65.2%)	78 (67.8%)	153 (66.5%)		0.176
Yes	40 (34.8%)	37 (32.2%)	77 (33.5%)		
Comorbidities					
Hypertension*				<0.001	
Non-hypertensive	94(81.7%)	113(98.3%)	207(90%)		6.231
Hypertensive	21(18.3%)	2(1.7%)	23(10%)		
Hypothyroid*				0.020	
No	99(86.1%)	110(95.7%)	209(90.9%)		6.341
Yes	16(13.9%)	5(4.3%)	21(9.1%)		

The basic laboratory data yielded significant differences with respect to haemoglobin, total cholesterol, high-density lipoprotein (HDL) and glycaemic indices, FBS, PPBS, and HbA1C (Table 2).

**Table 2:** Laboratory parameters among cases and control Continuous variables are expressed as mean (± standard deviation) and p-values based on the t-test and median (IQR) for the Wilcoxon-Mann-Whitney-U test. * marked values have p-values <0.05, which is a statistically significant value. Values for total cholesterol, HDL, VLDL, triglyceride, FBS, PPBS, and HbA1c were calculated using the Wilcoxon-Mann-Whitney-U test and haemoglobin was calculated using the t-test. HDL: high-density lipoprotein, VLDL: very low-density lipoprotein, FBS: fasting blood sugar, PPBS: postprandial blood sugar and HbA1c: glycated haemoglobin.

Characteristic	Cases n = 115	Matched Controls n = 115	Significance (p-value)	Test Value
Haemoglobin* (g/dL)	12.25 (± 1.61)	13.08 (±1.08)	<0.001	-4.562
Total Cholesterol* (mg/dL)	210(191-233.5)	179(158-202)	<0.001	9867.500
HDL* (mg/dL)	36(32-40)	44(42-50)	<0.001	1960.000
VLDL (mg/dL)	33.8(30.8-37.6)	35(31-41)	0.176	5929.500
Triglycerides (mg/dL)	169(154-188)	172(152-200)	0.411	6197.500
FBS* (mg/dL)	136(129-151.5)	88(85-90.5)	<0.001	13221.000
PPBS* (mg/dL)	210(199.5-239.5)	113( 109-118)	<0.001	13225.000
HbA1c* (%)	7.6(7.1-8.1)	5.1(4.9-5.3)	<0.001	13225.000

The platelet parameters yielded significant differences between the cases (diabetics) and controls (age and sex-matched healthy controls). The platelet count was decreased while IPF, MPV, PDW, PCT, platelet large cell count (PLCC), and platelet large cell ratio (PLCR) were increased among the cases (Table 3).

**Table 3:** Mean and median of platelet parameters Mean and median values for platelet count, IPF, MPV, PDW, PCT, PLCC, and PLCR. Data in parentheses indicate IQR. IQR: Interquartile range, IPF: immature platelet fraction, MPV: mean platelet volume, PDW: platelet distribution width, PCT: plateletcrit, PLCC: Platelet large cell count, PLCR: platelet large cell ratio. *Indicates the p-value less than 0.05 significance level. All values were calculated using the Wilcoxon-Mann-Whitney-U test.

Parameter	Median (IQR)		p-value	Test Value
	Cases N=115	Controls N=115		
Platelet Count (/microlitre)	208 (185.5-284.5)	242 (210.5-285.5)	0.002	5060.000
IPF (%)	15.7 (12.1-17.9)	6.3 (3.9-7.8)	<0.001	12438.500
MPV (fL)	17.2 (14.3-18.35)	11 (10.05-11.9)	<0.001	12562.000
PDW (%)	17.9 (16.4-19.4)	16.1 (15.2-16.5)	<0.001	11062.000
PCT (%)	0.3 (0.29-0.32)	0.26 (0.24-0.27)	<0.001	12408.500
PLCC (10^9^/L)	101 (97.7-110)	76 (65-85)	<0.001	12176.000
PLCR (%)	49.51 (42.64-53.72)	29.8 (24.72-34.32)	<0.001	15.517

**Figure 1:**
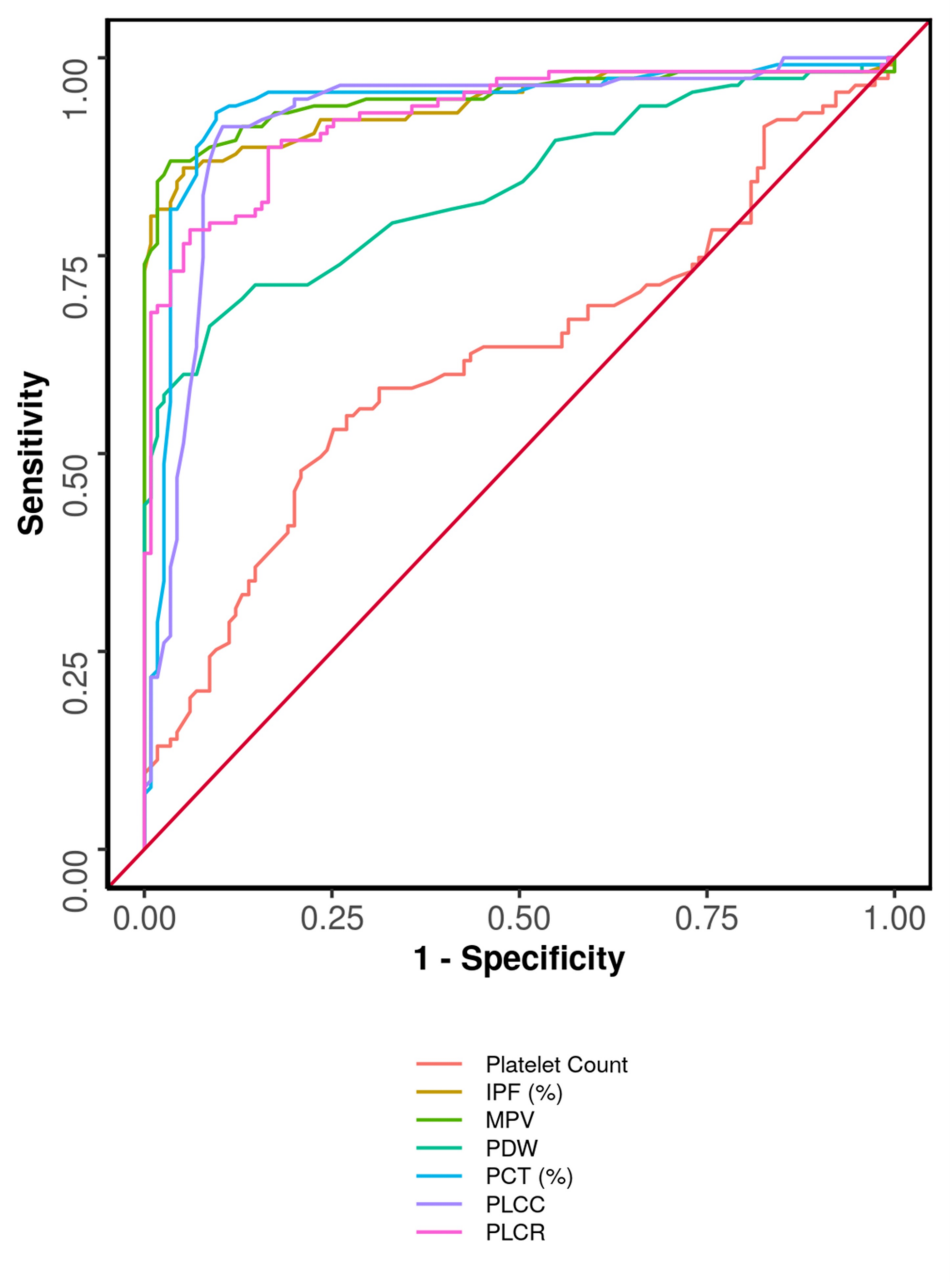
Comparison of the ROC of platelet parameters in predicting case vs control The figure depicting the ROC (receiver operator characteristic) curve for various platelet parameters. IPF: immature platelet fraction, MPV: mean platelet volume, PDW: platelet distribution width, PCT: plateletcrit, PLCC: platelet large cell count, PLCR: platelet large cell ratio.

Ours is a novel study which yielded information regarding cutoff values with sensitivity and specificity (Table 4). The parameter with the highest sensitivity was PCT, with a sensitivity of 93% at a cutoff of >0.282%, and the parameter with the highest specificity was MPV, with a specificity of 96.5% at a cutoff of >13.1 fL. Both parameters also demonstrated the best diagnostic accuracy, reaching 91.7%. The area under the ROC curve (AUROC) for IPF (%) predicting case vs control was 0.941, thus demonstrating excellent diagnostic accuracy (90.4%). At a cutoff of IPF (%) ≥9.6, it predicts a case with a sensitivity of 86% and a specificity of 95% which was at par with MPV and PCT (Table 5 and Figure 1).

**Table 4:** Performance of platelet parameters Data in parentheses indicate the 95% confidence interval. ROC: Receiver operating characteristic curve, IPF: immature platelet fraction, MPV: mean platelet volume, PDW: platelet distribution width, PCT: plateletcrit, PLCC: platelet large cell count, PLCR: platelet large cell ratio.

Platelet Parameter	Sensitivity	Specificity	PPV	NPV	Diagnostic Accuracy
Platelet Count (Cutoff: 209 by ROC)	53.0% (44-62)	74.8% (66-82)	67.8% (57-77)	61.4% (53-70)	63.9% (57-70)
IPF (%) (Cutoff: 9.6 by ROC)	86.1% (78-92)	94.8% (89-98)	94.3% (88-98)	87.2% (80-93)	90.4% (86-94)
MPV (fL) (Cutoff: 13.1 by ROC)	87.0% (79-93)	96.5% (91-99)	96.2% (90-99)	88.1% (81-93)	91.7% (87-95)
PDW (%) (Cutoff: 16.9 by ROC)	66.1% (57-75)	91.3% (85-96)	88.4% (80-94)	72.9% (65-80)	78.7% (73-84)
PCT (%) (Cutoff: 0.282 by ROC)	93.0% (87-97)	90.4% (84-95)	90.7% (84-95)	92.9% (86-97)	91.7% (87-95)
PLCC (10^9^/L) (Cutoff: 94 by ROC)	91.3% (85-96)	89.6% (82-94)	89.7% (83-95)	91.2% (84-96)	90.4% (86-94)
PLCR (%) (Cutoff: 41.991 by ROC)	78.3% (70-85)	93.9% (88-98)	92.8% (86-97)	81.2% (74-87)	86.1% (81-90)

**Table 5:** Most efficient platelet parameters AUROC: Area under the receiver operator characteristic curve, PPV: positive predictive value, NPV: negative predictive value, IPF: immature platelet fraction, MPV: mean platelet volume, PDW: platelet distribution width, PCT: plateletcrit, PLCC: platelet large cell count, PLCR: platelet large cell ratio.

Best Parameter in Terms of	Name of the Parameter
AUROC	MPV (fL) [0.95 (0.918 - 0.981)]
Sensitivity	PCT (%) (93%) at cutoff > 0.282 %
Specificity	MPV (fL) (96.5%) at cutoff > 13.1 fL
PPV	MPV (fL) (96.2%) at cutoff > 13.1 fL
NPV	PCT (%) (92.9 %) at cutoff > 0.282 %
Diagnostic accuracy	MPV (fL), PCT (%) (91.7%) at cut off >13.1 fL and >0.282%, respectively

**Figure 2:**
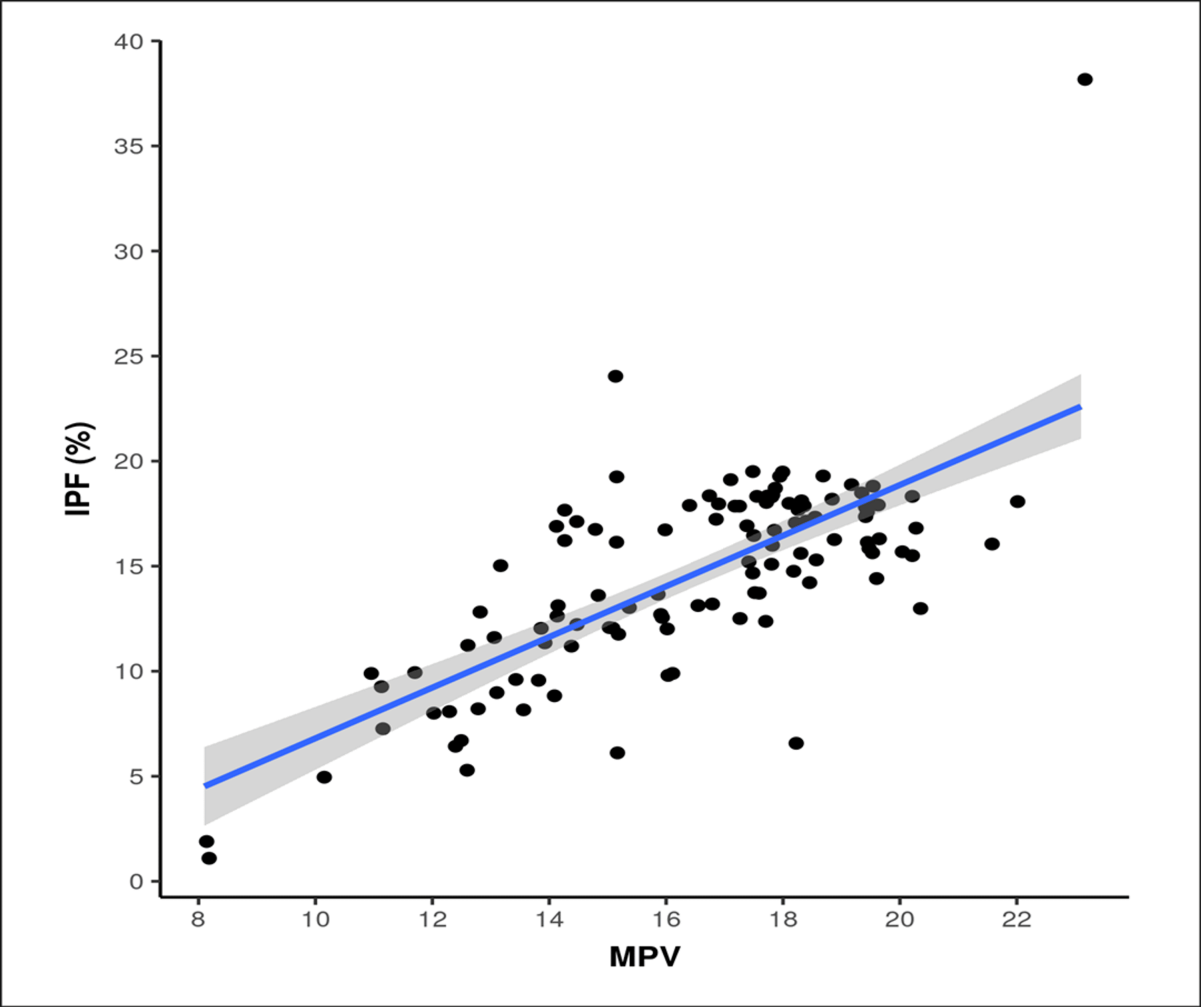
Scatter plot between IPF (%) and MPV (fL) The scatter plot between the MPV (mean platelet volume) and IPF (immature platelet fraction) showing a linear relationship.

There was a weak positive correlation between age (years) and IPF (%) and this correlation was statistically significant (rho = 0.26, p = 0.006). For every one-year increase in age, in the 30-59-year age group, IPF (%) increased by 0.16 %. There was a moderate negative correlation between BMI (kg/m²) and IPF (%) and this correlation was statistically significant (rho = -0.43, p <0.001). For every one kg/m^2^ increase in BMI, the IPF (%) decreased by 0.60%. No significant correlation was found between IPF and gender (male or female), smoking and dietary preferences (vegetarian or non-vegetarian). There was a moderate positive correlation between total cholesterol (mg/dL) and IPF (%) and this correlation was statistically significant (rho = 0.34, p <0.001). For every 1 mg/dl increase in total cholesterol (mg/dL), the IPF (%) increases by 0.04 %. There was a weak negative correlation between platelet count and IPF (%) and this correlation was statistically significant. There was a strong positive correlation between MPV and IPF (%) and this correlation was statistically significant (rho = 0.64, p = <0.001). For every 1 fL increase in MPV, the IPF (%) increases by 1.21%. Conversely, for every one percent increase in IPF (%), the MPV increases by 0.44 fL (Figure 2).

There was a moderate positive correlation between PDW and IPF (%) and this correlation was statistically significant (rho = 0.32, p <0.001). There was a moderate negative correlation between PCT (%) and IPF (%) and this correlation was statistically significant (rho = -0.33, p <0.001). No significant correlation was found between IPF and PLCC and between IPF and PLCR.

## Discussion

DM is a multifaceted metabolic disorder marked by persistent hyperglycaemia, which can lead to complications involving the peripheral nerves, kidneys, and eyes, as well as microvascular and macrovascular systems. At the cellular level, platelets are one of the components which are significantly affected by the disease process. Data suggest myriad pathophysiological mechanisms by which diabetes influences various blood cell lines. In diabetic patients, chronic hyperglycaemia triggers the production of reactive oxygen species, leading to oxidative stress and endothelial dysfunction. It also promotes platelet hyperactivity by causing glycation of surface proteins and increasing the expression of glycoprotein IIb/IIIa (GP IIb/IIIa) and P-selectins due to hyperglycaemia-induced hyperosmolarity. Moreover, hyperglycaemia is associated with higher levels of von Willebrand factor (vWF), tissue factor, and reduced fibrinolysis, all of which contribute to the procoagulant state of diabetes [3]. Kelem et al. studied insulin resistance-induced platelet hyperactivity and the role of platelet parameters as a potential biomarker. Studies have shown that platelets have insulin receptors which in turn regulate platelet action [4].

This study provides valuable insights into the relationship between platelet parameters and T2DM, particularly focusing on IPF, MP, and PCT. Our findings indicate that MPV and PCT are highly effective markers for assessing platelet dynamics in the diabetic population. MPV, with a cutoff of ≥13.1 fL, showed exceptional specificity (96.5%) and the highest positive predictive value (96.2%), making it a strong candidate for identifying prothrombotic states in diabetes. PCT, on the other hand, demonstrated the highest sensitivity (93%) at a cutoff >0.282% and the best negative predictive value (92.9%), highlighting its utility as a sensitive screening tool.

In a study by Lee et al., the IPF was found to be significantly elevated which continued to remain significant after logistic regression [5]. These results are similar to our study and reinforce the use of IPF for utilization as a marker for the prothrombotic state in diabetes. Interestingly, in a study by Verdoia et al., no significant difference was observed in IPF between diabetics and non-diabetics [6]. This study was conducted in patients with coronary artery disease undergoing coronary angiography. The contrasting finding was attributed to the lack of healthy controls for comparison in this study. Astuti et al. did an analytical observational study to determine the relationship between IPF, MPV and type 2 DM. They stated that diabetes mellitus was a condition promoting atherothrombosis through poor glycaemic control which led to adverse cardiac events. The glycation of platelet surface proteins led to an enhanced platelet activation and response to stimuli. The platelet aggregation was stimulated. This led to an increased platelet turnover and the formation of new, immature platelets [7]. Even though a smaller sample size limited significant results demonstrating relations between blood sugar levels and IPF, these studies lay a foundation for future studies. 

We observed that the mean MPV (fL) among the cases was 16.44 (± 2.87) and among the controls was 11.04 (± 1.19). The difference was statistically significant. In a previous study by Lee et al., a similar relation was found [5]. Astuti et al. demonstrated a similar relationship between diabetes and MPV. The higher MPV values were associated with poorer glycaemic control and worse outcomes [7]. A meta-analysis conducted by Zaccardi et al. also shows that the MPV of people with type 2 DM is significantly higher compared to healthy individuals [8]. In our study, we observed that the mean PDW among the cases was 18.09 (±2.29) and among the controls was 15.72 (± 1.21) which had a statistical significance. Similarly, in a cross-sectional study by Demirtas et al. PDW was found to be significantly elevated among the subgroups with higher HbA1c values [9]. This larger variation in platelet size could be due to heightened platelet activation and hyperglycaemia-associated endothelial dysfunction, oxidative stress and inflammatory state.

We observed significantly higher values of all the platelet parameters in the diabetic subgroup. Among these parameters, we observed that MPV was the most specific and PCT was the most sensitive, although all the other parameters were also significantly higher among diabetics.

IPF was found to be significantly higher in the diabetes subgroup. It was found to be superior to the platelet count and PDW as an analytic parameter for predicting diabetes and its complications. We further go on to propose a cutoff of IPF (%) ≥9.6, with a sensitivity of 86% and a specificity of 95%. This study is the first of its kind to propose such cutoffs. These studies could be used as stepping stones for larger cohorts to arrive at a significant cutoff value for the Indian population in the future. In addition to comparing IPF between cases and controls, we examined its relationship with other parameters in all 230 participants and found a significant positive relationship between IPF and age within the 30-59 years age group. In our study, we observed a negative correlation between IPF and BMI, which contrasts with the findings reported by Goudswaard et al. In their study, they found that higher BMI correlated with an increase in immature platelet count [10]. This could be attributed to several factors, including the relatively limited sample size and the narrow range of BMI variations observed in our study. IPF was found to have a positive correlation with total cholesterol levels. This association may be explained by the known effect of hypercholesterolemia on platelet priming and activation [11]. Our study found no correlation between IPF and glycaemic indices, such as fasting blood sugar, postprandial blood sugar, and HbA1c, consistent with findings by Verdoia et al. However, drawing definitive conclusions from cross-sectional data is challenging, as serial readings may be necessary to understand IPF dynamics with changing glycaemic levels. In contrast, Lee et al. observed a significant association between higher HbA1c and increased IPF, but their study included patients with longer diabetes duration and pre-existing complications, which were excluded from our study [5]. These differences might also explain why we found no correlation between IPF and treatment modalities, unlike Lee et al. who observed a decrease in IPF with intensified treatment. The complexity of the relationship between IPF, glycaemic control, and treatment strategies highlights the need for further long-term prospective research.

We also observed IPF to have a significant correlation with platelet count, MPV, PDW and PCT. Our analysis showed a negative correlation with a 1000/mL increase in platelet leading to a fall in IPF by 0.02 % and vice versa. IPF has been studied in thrombocytopenic conditions, where an increased peripheral destruction of platelets leads to an increased production by the bone marrow and an earlier release of immature platelets. With diabetes providing an environment for peripheral activation and utilization, similar mechanisms could be contributing to the negative correlation between IPF and platelet count observed in our study. A strong positive correlation was observed in our study between IPF and MPV. In our case-control study, we found significantly higher values of MPV, PDW, PLCC and PLCR in the diabetic subgroup. In our study, MPV and PCT were the most specific and sensitive parameters for assessing the diabetic population. We recommend an MPV cutoff of ≥13.1 (87% sensitivity, 96% specificity) and a PCT cutoff of >0.282 (93% sensitivity, 90.4% specificity). MPV proved more effective than PDW and platelet count. These readily accessible metrics in routine lab reports can offer valuable insights into platelet dynamics without additional effort.

Although the IPF has been widely utilized in thrombocytopenia and other haematological diseases, its role in diabetes has not been thoroughly investigated. Our study explores the relationship between IPF and diabetes, alongside other platelet parameters, and proposes potential cutoff values to enhance its diagnostic and clinical relevance in this context. Exact age and sex matching of cases and controls alleviates any selection bias thereby further reinforcing the higher IPF values in diabetics. IPF along with other indices could assist in the early identification of the development of diabetic complications and enable an earlier intervention to reduce the morbidity associated with the disease.

Our study contributes valuable insights into the relationship between platelet indices and diabetes, potentially paving the way for future research and clinical applications aimed at improving patient outcomes in the management of this prevalent disease. Conducting a multi-center prospective cohort study could provide deeper insights into this association. Additionally, extending the duration of follow-up, particularly in individuals with a family history or higher risk for diabetes, could enhance the comprehensiveness of our findings. Interventions aimed at modulating platelet activity and mitigating the prothrombotic state associated with diabetes may hold promise for preventing or delaying the onset of microvascular and macrovascular complications in affected individuals.

Limitations

Being a single-centre case-control study, our results may not be generalizable to a larger population. Despite careful selection, there were statistically significant differences in comorbidities, such as hypertension and hypothyroidism, between the cases and controls, though these were limited in number to draw any inferences.

## Conclusions

This study highlights the significant relationship between platelet indices and T2DM, with a particular focus on the IPF as a promising marker to predict individuals at an increased risk of developing diabetic complications in future. Our findings suggest that these platelet parameters could be valuable in identifying prothrombotic states early in diabetes, which may aid in preventing complications. Future larger cohort studies are necessary to validate the proposed cutoff values and explore their clinical implications, especially in diverse populations. These insights can potentially lead to earlier interventions and improved management of T2DM, thereby reducing its associated morbidity.

## References

[1] Sun H, Saeedi P, Karuranga S (2022). IDF Diabetes Atlas: Global, regional and country-level diabetes prevalence estimates for 2021 and projections for 2045. Diabetes Res Clin Pract.

[2] (2024). 2. Diagnosis and classification of diabetes: standards of care in diabetes-2024. Diabetes Care.

[3] Kaur R, Kaur M, Singh J (2018). Endothelial dysfunction and platelet hyperactivity in type 2 diabetes mellitus: molecular insights and therapeutic strategies. Cardiovasc Diabetol.

[4] Kelem A, Adane T, Shiferaw E (2023). Insulin resistance-induced platelet hyperactivity and a potential biomarker role of platelet parameters: a narrative review. Diabetes Metab Syndr Obes.

[5] Lee EY, Kim SJ, Song YJ, Choi SJ, Song J (2013). Immature platelet fraction in diabetes mellitus and metabolic syndrome. Thromb Res.

[6] Verdoia M, Nardin M, Rolla R (2020). Impact of diabetes mellitus on immature platelet fraction and its association with coronary artery disease. Diabetes Metab Res Rev.

[7] Astuti DW, Wibisono S, Hajat A, Soehita S (2017). Correlations between mean platelet volume and immature platelet fraction to hemoglobin A1C in patients with type 2 diabetes mellitus. Indones J Clinical Pathol Med Laboratory.

[8] Zaccardi F, Rocca B, Pitocco D, Tanese L, Rizzi A, Ghirlanda G (2015). Platelet mean volume, distribution width, and count in type 2 diabetes, impaired fasting glucose, and metabolic syndrome: a meta-analysis. Diabetes Metab Res Rev.

[9] Demirtas L, Degirmenci H, Akbas EM, Ozcicek A, Timuroglu A, Gurel A, Ozcicek F (2015). Association of hematological indicies with diabetes, impaired glucose regulation and microvascular complications of diabetes. Int J Clin Exp Med.

[10] Goudswaard LJ, Corbin LJ, Burley KL (2022). Higher body mass index raises immature platelet count: potential contribution to obesity-related thrombosis. Platelets.

[11] Wang N, Tall AR (2016). Cholesterol in platelet biogenesis and activation. Blood.

